# Combinatorial Effects of Free and Nanoencapsulated Forms of Cabazitaxel and RAS-Selective Lethal 3 in Breast Cancer Cells

**DOI:** 10.3390/pharmaceutics17050657

**Published:** 2025-05-17

**Authors:** Remya Valsalakumari, Marek Feith, Solveig Pettersen, Andreas K. O. Åslund, Ýrr Mørch, Tore Skotland, Kirsten Sandvig, Gunhild Mari Mælandsmo, Tore-Geir Iversen

**Affiliations:** 1 Department of Tumor Biology, Institute for Cancer Research, Oslo University Hospital, 0379 Oslo, Norway; remyav@uio.no (R.V.); marek.feith@studmed.uio.no (M.F.); g.m.malandsmo@ous-research.no (G.M.M.); 2 Department of Molecular Cell Biology, Institute for Cancer Research, Oslo University Hospital, 0379 Oslo, Norway; tore.skotland@ous-research.no (T.S.); kirsten.sandvig@ibv.uio.no (K.S.); 3 Institute of Clinical Medicine, Faculty of Medicine, University of Oslo, 0379 Oslo, Norway; 4 Department of Biotechnology and Nanomedicine, SINTEF AS, 7034 Trondheim, Norway; andreas.aaslund@sintef.no (A.K.O.Å.); yrr.morch@nadeno.com (Ý.M.); 5 NaDeNo Nanoscience AS, 7051 Trondheim, Norway; 6 Centre for Cancer Cell Reprogramming, Faculty of Medicine, University of Oslo, 0379 Oslo, Norway; 7 Department of Biosciences, University of Oslo, 0316 Oslo, Norway; 8 Department of Medical Biology, University of Tromsø, 9019 Tromsø, Norway

**Keywords:** cabazitaxel, RSL3, combinatorial effects, breast cancer, poly(2-ethyl butyl cyanoacrylate), nanoparticles

## Abstract

**Background:** Combination therapies for cancer have gained considerable attention due to their potential for enhancing therapeutic efficacy and decreasing drug resistance. Introducing nanodrug delivery systems in this context may further improve the therapy due to targeted delivery, improved drug stability, sustained drug release, and prevention of rapid clearance from circulation. This study evaluates the combinatorial effects of two cytotoxic drugs, cabazitaxel (CBZ) and RSL3 (RAS-selective lethal 3), in free form as well as encapsulated within poly(2-ethyl butyl cyanoacrylate) (PEBCA) nanoparticles (NPs) in breast cancer cell lines. **Methods**: Cell proliferation was assessed using IncuCyte technology, and synergistic drug effects were determined with SynergyFinder Plus. Cell viability was measured with the MTT assay. Additionally, we investigated whether the combinatorial effects were reflected in alterations of metabolic activity or reactive oxygen species (ROS) production using Seahorse technology and the CM-H_2_DCFDA assay, respectively. **Results**: The data presented reveal, for the first time, that CBZ and RSL3 exhibit synergistically or additively combinatorial effects on various breast cancer cell lines. The pattern of cytotoxic effects was consistent, whether the drugs were in free form or encapsulated in NPs. Moreover, the combinatorial effects were not observed to be associated with early changes in metabolic activity or ROS production. **Conclusion**: This study highlights the potential of CBZ and RSL3 in combinatorial nanomedicine as they may act synergistically. Further studies are warranted to better understand the mechanisms behind these combinatorial effects.

## 1. Introduction

Cancer is a multifactorial disease, where different cells and diverse molecules are engaged in complex regulatory networks and cross-talks between biological processes [1,2,3]. Hence, it is poorly manageable with a single therapeutic agent, which may lead to drug resistance [4] and tumor recurrence [5]. Combination therapy involving two or more therapeutic agents or modalities has evolved as a key strategy in cancer therapy [6]. Combining multiple agents may enhance efficacy, reduce drug resistance, and improve the overall survival of patients by targeting multiple pathways or mechanisms either synergistically or independently [6,7,8]. In the present scenario, chemotherapy has become an indispensable part of treatment even if the drugs used contribute to undesirable effects and adverse reactions [9]. This results in a narrow therapeutic window in addition to resistance [10]. Hence, combining chemotherapy with radiation, immunotherapy, or other targeted therapies has now been implemented in clinical practice, and novel combinations are continually being reported at the preclinical phase or undergoing clinical trials [11,12,13,14].

Cabazitaxel (CBZ) is a second-generation taxane approved for the treatment of castration-resistant prostate cancer [15] and has also undergone clinical evaluation for the treatment of breast cancer [16,17,18]. It is a microtubule inhibitor like other taxanes but has the advantage of having less affinity to P-glycoprotein, resulting in increased cellular retention and higher efficacy [19]. Still, low aqueous solubility and toxicity can limit clinical use, where alternate approaches are needed [20]. RSL3 is a known ferroptosis inducer [21], and it has been reported to induce improved anticancer effects when combined with other chemotherapy drugs, monoclonal antibodies, and mTOR inhibitors through different mechanisms [22,23,24,25]. It has been reported that RSL3 acts by inactivating the protein glutathione peroxidase 4 (GPX4) after binding to its active site, which in turn causes iron-dependent accumulation of ROS within the cells and eventually leads to ferroptotic cell death [21]. However, poor pharmacokinetic properties [26] and off-target side effects limit its potential for clinical applications.

In the present study, we have investigated how the combination of CBZ and RSL3 affects the viability of breast cancer cell lines. These cell lines, SKBR3, JIMT-1 and MCF7 (all human), and EO771 (murine), have different expression levels of estrogen receptors (ER), progesterone receptors (PR) and HER2 receptors, and they also have different sensitivity to CBZ and RSL3.

The effects were evaluated either when the drugs were in their free form or encapsulated within PEGylated PEBCA NPs. PEBCA NPs are synthesized by polymerization of 2-(ethyl butyl cyanoacrylate) monomers and possess excellent properties to encapsulate hydrophobic molecules such as CBZ or RSL3. We have published multiple articles evaluating the biological effects and efficacy of these NPs loaded with CBZ in different models of breast cancer, which showed promising results; see, e.g., [27,28,29]. Hence, it was interesting to conduct studies comparing the combinatorial effects of drugs in their free form or encapsulated within the PEBCA NPs. The present study demonstrates that CBZ and RSL3 act synergistically and are promising candidates for developing combinatorial therapy for breast cancer. Nonetheless, the mechanism of action needs further elucidation. Moreover, encapsulation of these drugs within PEBCA NPs did not hamper the combinatorial effects, indicating that these NPs may improve the pharmacokinetic properties and thus should be studied further in in vivo models.

## 2. Materials and Methods

### 2.1. Materials

RPMI 1640 medium, DMEM medium, bovine serum albumin (BSA), fetal bovine serum (FBS), dimethyl sulfoxide (DMSO), 3-(4,5-dimethyl-2-thiazolyl)-2,5-diphenyltetrazolium bromide (MTT; cat#M5655), 1S,3R-RSL3 (https://www.sigmaaldrich.com/NO/en/product/sigma/sml2234 (accessed on 24 August 2022); info of, i.e., formula and purity, >98%) and penicillin/streptomycin (Pen/Strep P4333) were purchased from Merck (Darmstadt, Germany). DMEM F12 medium was procured from ThermoFisher Scientific (Waltham, MA, USA). CBZ was obtained from Biochempartner Co., Ltd. (Shanghai, China, https://www.biochempartner.com/183133-96-2-BCP27641 (accessed on 24 August 2022); i.e., with information on formula and purity, 98%). Milli-Q water was freshly prepared by the Millipore Milli-Q Biocell purification system (Burlington, MA, USA). All chemicals used were of analytical quality. Materials used for NP synthesis and other reagents are mentioned together with the methods.

### 2.2. Cell Lines

A panel of breast cancer cell lines having different molecular characteristics was used for the study. EO771 cells (murine, ER^α−^ PR^+^, HER2^+^) were cultured in DMEM. SKBR3 (ER^−^, PR^−^, HER2^+^) and MCF-7 (luminal A, ER^+^, PR^+^, HER2^−^) cells were cultured in RPMI 1640. JIMT-1 cells (ER^−^, PR^−^, HER2^+^) were cultured using a 1:1 mixture of DMEM/F-12 medium. All media were supplemented with 10% (*v*/*v*) FBS and 100 units/mL penicillin/streptomycin. JIMT-1 cells were obtained from the German Collection of Microorganisms and Cell Cultures (DSMZ; Braunschweig, Germany). All cells were routinely subjected to mycoplasma testing.

### 2.3. Synthesis and Characterization of NPs

PEGylated PEBCA NPs were synthesized using a miniemulsion polymerization technique [27,28,29,30]. An oil phase (2.7 mL), containing 0.9 g of 2-ethylbutyl cyanoacrylate (EBCA) (Cuantum Medical Cosmetics, Bellaterra, Spain) and the co-stabilizer Miglyol^®^ 812 (1.1% (*w*/*w*), Cremer Oleo GmbH & Co., KG, Hamburg, Germany), was mixed with a water phase (6.5 mL) consisting of the non-ionic PEG stabilizers Brij^®^ L23 (5 mM, Sigma-Aldrich, St. Louis, MO, USA) and Kolliphor^®^ HS 15 (6 mM, Sigma-Aldrich) in 0.1 M HCl. Compounds to be encapsulated were added to the oil phase (CBZ, 10% (*w*/*w* oil phase), and RSL3, 10% (*w*/*w* oil phase)) prior to mixing. The oil phase was added to the aqueous phase and immediately sonicated for 3 min on ice (6 × 30 sec intervals, 60% amplitude, Branson Ultrasonics digital sonifier 450, Brookfield, CT, USA). The solution was then rotated (15 rpm, Stuart SB3 rotator, Keison Products, Chelmsford, UK) at room temperature (RT) over two nights. The emulsions were extensively dialyzed (Spectra/Por dialysis membrane MWCO 100,000 Da, Spectrum Labs, Rancho Dominguez, CA, USA) against 1 mM HCl (pH 3.0). Loading of CBZ within PEBCA NPs was quantified by mass spectrometry analysis (Agilent, Santa Clara, CA, USA, 1290 HPLC system coupled to an Agilent 6490A triple quadrupole mass spectrometer), and encapsulation efficiency was determined as described earlier [27]. Loading of RSL3 was quantified by mass spectroscopy analysis. RSL3-PEBCA NPs were diluted in DMSO/acetonitrile/ammonium acetate (50%/45%/5%), and separation was performed using a column (Ascentis Express Phenyl Hexyl) with mobile phases A: water with 25 mM formic acid and B: acetonitrile with 25 mM formic acid. The size (diameter; z-average: intensity-based mean calculations), polydispersity index (PDI), and zeta potential (calculations based on the Helmholtz-Smoluchowski equation) of the NPs were measured in PBS, pH 7.4 (diluted 1:50, approx.NP conc. 2 µg/mL) by dynamic light scattering using a Zetasizer Nano ZS Malvern Instrument and the accompanied software package (ver. 6.01) for data acquisition and analysis (Malvern, UK). The NPs were found to be stable regarding size, drug loading and colloidal stability for a period of 2 years when stored at 4 °C in 1 mM HCl (pH 3.0), during which the experiments were performed.

### 2.4. Evaluation of Cell Proliferation

Cellular proliferation was evaluated by measuring cell confluence using the Incucyte technology [31]. Briefly, cells were seeded at a density of 8 × 10^3^ cells/well of 96-well plates in suitable medium (200 µL). The plates were incubated for 24 h before treating them with either free drugs, PEBCA drugs or combinations in triplicate. This is followed by real-time image-based cell density measurements using the Incucyte^®^ S3 live cell imaging and analysis system (Sartorius, Gottingen, Germany) equipped with a confluence analysis module. At least three images were captured per well. Measurements were started on day 0 of treatment and continued up to 4–6 days, depending upon the cell line. Data were plotted as % cell confluence normalized to the day of treatment initiation.

### 2.5. Analysis of Combinatorial Effects

The cell density measurements retrieved from the Incucyte analysis were used to assess the combinatorial effects of the treatments using the SynergyFinder Plus (version 3.10.3) web application [32]. Briefly, the normalized values of cell density measurements during the last time point of treatment were used to calculate percent inhibition with respect to untreated control. The SynergyFinder Plus application used these values to calculate the synergy scores according to different reference models. The degree of synergistic or antagonistic effect is quantified by comparing the observed combinatorial response against the expected response, calculated using a reference model that assumes no interaction between the drugs. Different reference models quantify the degree of synergy either as the excess over the maximum single drug response (highest single agent (HSA)) [33], as the multiplicative effect of single drugs as if they acted independently (Bliss) [34], as the expected response corresponding to an additive effect as if the single drugs were the same compound (Loewe) [35], or as the expected response corresponding to the effect as if the single drugs did not affect the potency of each other (zero interaction potency (ZIP)) [36]. Suitable synergy models were chosen for each cell line, according to the observed drug responses. The threshold is set in a way that synergy scores greater than 10 indicate synergy, scores between −10 and +10 indicate additivity and scores less than −10 indicate no combinatorial effects. However, synergistic effects are highly contextual; hence, this scale should be assumed as a spectrum where increasing positive values from zero span from additivity towards synergy and negative values from zero span across additivity towards no effect and then towards antagonistic effect.

### 2.6. Cell Viability Assay

Cell viability was assessed by the MTT assay as described elsewhere [29]. Briefly, cells were seeded and treated as described for the proliferation assay. Treated plates were incubated for 96 h at 37 °C. The medium was then aspirated, followed by the addition of MTT reagent (100 µL of 0.25 mg/mL in serum-free, phenol red-free medium) and further incubation for 3 h at 37 °C. Afterwards, the formazan crystals formed were dissolved in DMSO with 0.5% ammonia solution (200 µL/well), and the absorbance was measured at 570 nm against a 650 nm reference wavelength using a Synergy2 microplate reader (Biosys Ltd., Essex, UK). Percent cell viability was calculated with respect to the untreated control.

### 2.7. ROS Assay

Cells were seeded in glass-bottom white plates (Costar, Corning, NY, USA) at a density of 1.5 × 10^4^/well in 200 µL media. After 24 h, the cells were gently washed with 100 µL pre-warmed medium without FBS and phenol red, followed by the addition of 10 µM ROS sensor (General Oxidative Stress Indicator, CM-H_2_DCFDA, Invitrogen; https://assets.thermofisher.com/TFS-Assets/LSG/manuals/mp36103.pdf (accessed on September 1 2022)) (50 µL/well). The plate was then incubated in the dark at 37 °C for 45 min. Subsequently, the sensor was removed, and the cells were treated with indicated concentrations of free drugs, PEBCA drugs, combinations or empty PEBCA NPs in suitable media. H_2_O_2_ (500 µM) was used as a positive control. Fluorescence readout was performed after 1 h and 5 h of treatment using a Victor™ X3 multiplate reader (Perkin Elmer, Waltham, MA, USA) equipped with fluorescent filters (excitation 485 nm, emission 535 nm) and a prewarmed plate holder at 37 °C. Data of treated cells are normalized to cells treated only with the sensor.

### 2.8. Metabolic Assay

Mitochondrial metabolism was assessed using the Seahorse XFe96 Analyzer (Agilent, Santa Clara, CA, USA) employing the Mito Stress Test according to the manufacturer’s guidelines [37]. Briefly, the assay cartridge was hydrated and placed in an incubator at 37 °C without CO_2_ supplementation overnight. Cells were incubated in Seahorse 96-well plates overnight and treated with either free drugs, PEBCA drugs, combinations or empty PEBCA NPs for 4 h. The cells were then rinsed with phenol red and bicarbonate-free medium (pH 7.4), supplemented with 10 mM glucose, 1 mM pyruvate, and 2 mM L-glutamine (Agilent, Santa Clara, CA, USA), and incubated for 1 h at 37 °C without CO_2_. Modulating compounds used in the assay included 1.5 µM oligomycin, 0.5 µM rotenone/antimycin A (Agilent, Santa Clara, CA, USA), and 0.5 µM carbonyl cyanide 4-(trifluoromethoxy) phenylhydrazone (FCCP). Oligomycin inhibits ATP synthase, leading to a reduction in mitochondrial respiration by limiting the flow of electrons through the electron transport chain (ETC). FCCP, a protonophore, uncouples the proton gradient from respiration, enabling unrestricted electron flow through mitochondrial complexes and maximizing oxygen consumption at complex IV by disrupting the mitochondrial membrane potential. The spare respiratory capacity was determined as the difference between maximal and basal respiration rates. Additionally, the injection of rotenone and antimycin A inhibits complexes I and III, effectively halting the entire electron transport chain and allowing the measurement of non-mitochondrial respiration, driven by processes outside the mitochondria. The oxygen consumption rate (OCR) was measured using a solid-state sensor that detects changes in dissolved oxygen and free proton concentrations, which reflect oxygen consumption from mitochondrial respiration. Seahorse Wave Software v2.6.3 was utilized for data analysis. Normalization was conducted by determining total protein concentration via BCA Protein Assay (ThermoFischer Scientific, Waltham, MA, USA) at the end of the experiment, following the manufacturer’s instructions.

### 2.9. Statistical Analysis

Statistical analysis was performed using GraphPad Prism v9 (GraphPad Software, La Jolla, CA, USA). Statistical significance in the experiments was determined either by one-way ANOVA followed by the Tukey–Kramer multiple comparison test. In some cases, a two-tailed unpaired Student’s test was performed, which has been mentioned in respective figure legends. *p* values < 0.05 were considered statistically significant.

## 3. Results and Discussion

### 3.1. Physicochemical Characterization of NPs

**Figure 1:**
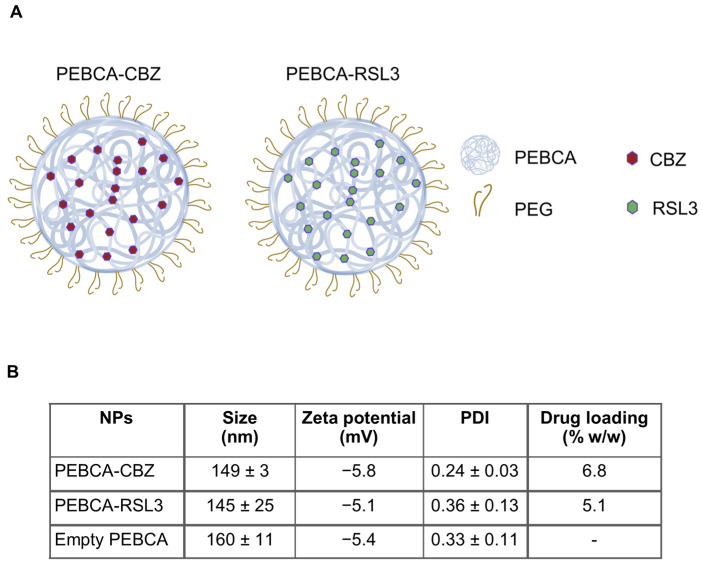
Schematic representation and physicochemical properties of NPs. (**A**) Representation of PEGylated PEBCA NPs used in different studies: empty NPs and NPs loaded either with CBZ or RSL3. (**B**) Physicochemical characteristics of NPs such as size, zeta potential, PDI and % (*w*/*w*) drug loading with respect to CBZ and RSL3. Size and PDI were measured whenever the NPs were used for experiments. Presented here are averages of 4 measurements performed from the time of preparation and for a period of 2 years. Zeta potential and drug loading were measured at one time point, within a week after the preparation of the NPs.

The NPs used and their physicochemical properties are shown in Figure 1. When combinatory treatment with PEBCA-CBZ and PEBCA-RSL3 is performed, the terminology PEBCA drugs is used. NPs exhibit a size range of 130–175 nm with PDI less than 0.36 in PBS, pH 7.4. They are also similar in their structure and PEGylation levels, while they differ in the percentage of drug loading, which is 6.8% and 5.1% of total dry weight for PEBCA-CBZ and PEBCA-RSL3, respectively.

### 3.2. Combination of CBZ and RSL3 Improved Cytotoxic Effects Compared to Drugs Alone

**Figure 2:**
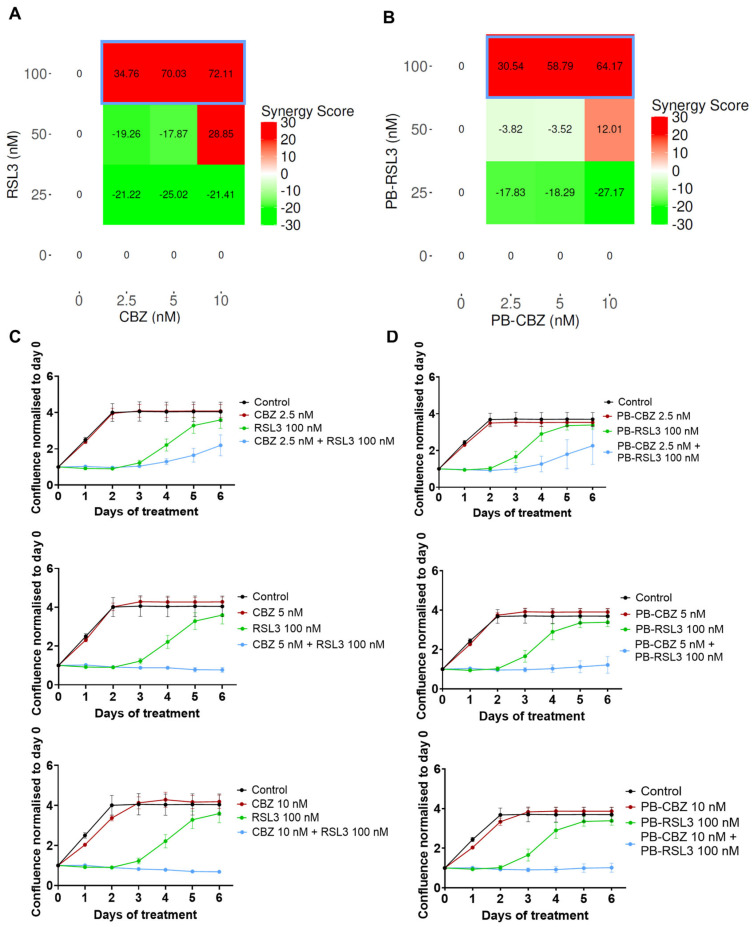
Combinatorial effects of CBZ and RSL3 or PEBCA-CBZ and PEBCA-RSL3 in EO771 cells. Figures shown in the left panel correspond to free drugs or their combinations, and the right panel corresponds to PEBCA drugs or their combinations. EO771 cells were treated with different concentrations of CBZ/PEBCA-CBZ and RSL3/PEBCA-RSL3 as single drugs or as combinations. Drug responses were evaluated by quantifying cell confluence through Incucyte analysis, and synergy scores were derived using SynergyFinder Plus. HSA model scores are shown here. (**A**,**B**) Synergy scores of all combinations used, respectively, for drugs alone and PEBCA drugs, confirming the synergistic effects of different concentrations of CBZ with higher concentrations of RSL3. Combinations showing high synergy scores are outlined in blue (**C**,**D**). Plots showing drug responses of combinations that showed synergistic effects and highest scores (outlined in blue in panels (**A**,**B**)), respectively, for drugs alone and PEBCA drugs. Presented here are mean values with standard deviations (error bars) of cell confluence normalized to day 0 of treatment from one out of three independent experiments, each run with triplicates. PEBCA is abbreviated to PB in graphs.

**Figure 3:**
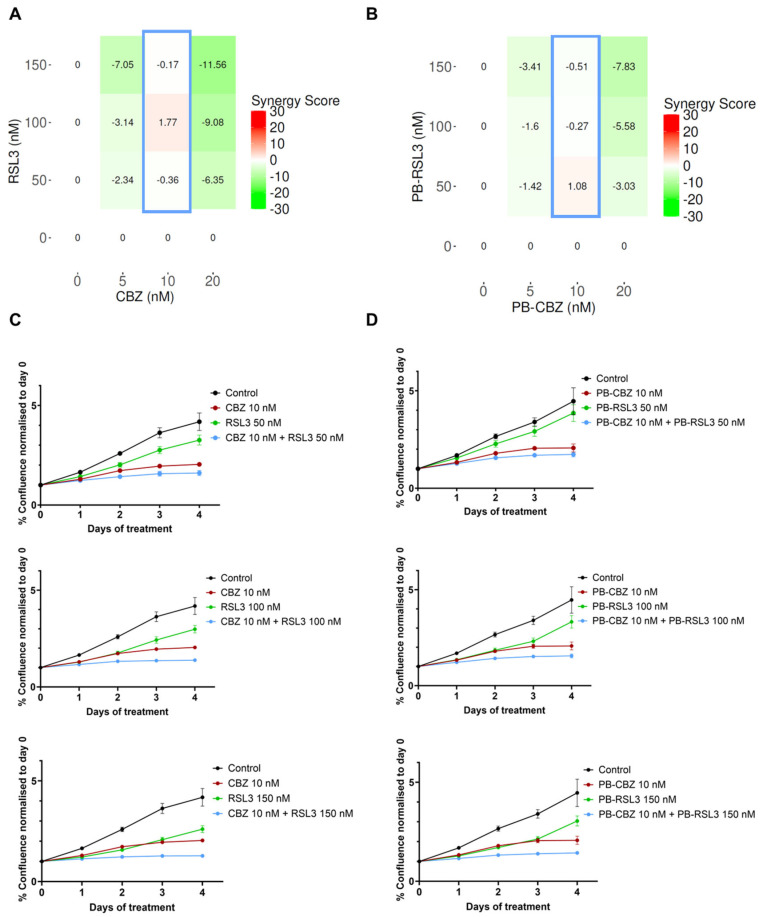
Combinatorial effects of CBZ and RSL3 or PEBCA-CBZ and PEBCA-RSL3 in SKBR3 cells. Figures shown in the left panel correspond to free drugs or their combinations, and the right panel corresponds to PEBCA drugs or their combinations. SKBR3 cells were treated with different concentrations of CBZ/PEBCA-CBZ and RSL3/PEBCA-RSL3 as single drugs or as combinations. Drug responses were evaluated by quantifying cell confluence through real-time Incucyte analysis for up to 4 days. Combinatorial effects were analyzed, and synergy scores were derived using SynergyFinder Plus software. Bliss model scores are shown here. (**A**) Synergy scores of all combinations used, respectively, for drugs alone and PEBCA-drugs, confirming the additive effects of certain combinations. Combinations showing the highest scores are outlined in blue. (**C**,**D**) Plots showing the drug responses of combinations that showed additive effects and highest scores (outlined in blue in panels (**A**,**B**)), respectively, for drugs alone and PEBCA drugs. Presented here are average values with standard deviations in error bars of % cell confluence over a period of 4 days from one out of three independent experiments run with triplicates. PEBCA is abbreviated to ‘PB’ in graphs.

**Figure 4:**
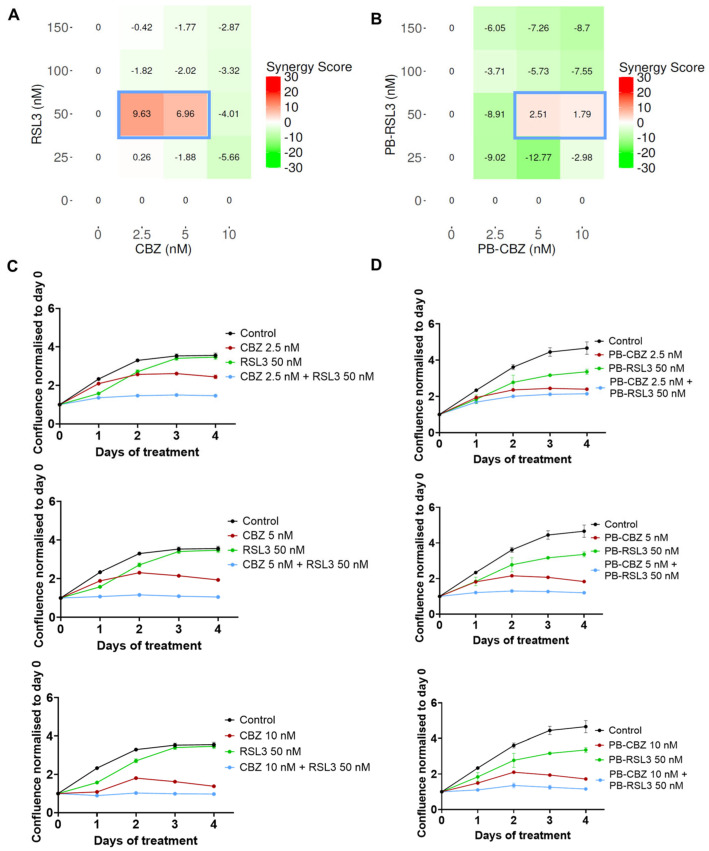
Combinatorial effects of CBZ and RSL3 or PEBCA-CBZ and PEBCA-RSL3 in JIMT-1 cells. Figures shown in the left panel correspond to free drugs or their combinations, and the right panel corresponds to PEBCA drugs or their combinations. JIMT-1 cells were treated with different concentrations of CBZ/PEBCA-CBZ and RSL3/PEBCA-RSL3 as single drugs or as combinations. Drug responses were evaluated by quantifying cell confluence through real-time Incucyte analysis for up to 4 days. Combinatorial effects were analyzed, and synergy scores were derived using SynergyFinder Plus software. Bliss model scores are shown here. (**A**,**B**) Synergy scores of all combinations used, respectively, for drugs alone and PEBCA drugs, confirming the additive effects of certain combinations. Combinations showing the highest scores are outlined in blue. (**C**,**D**) Plots showing drug responses of combinations that showed additive effects and highest scores (outlined in blue in panels (**A**,**B**)), respectively, for drugs alone and PEBCA drugs. Presented here are average values with standard deviation (error bars) of % cell confluence over a period of 4 days from one out of three independent experiments run with triplicates. PEBCA is abbreviated to PB in graphs.

**Figure 5:**
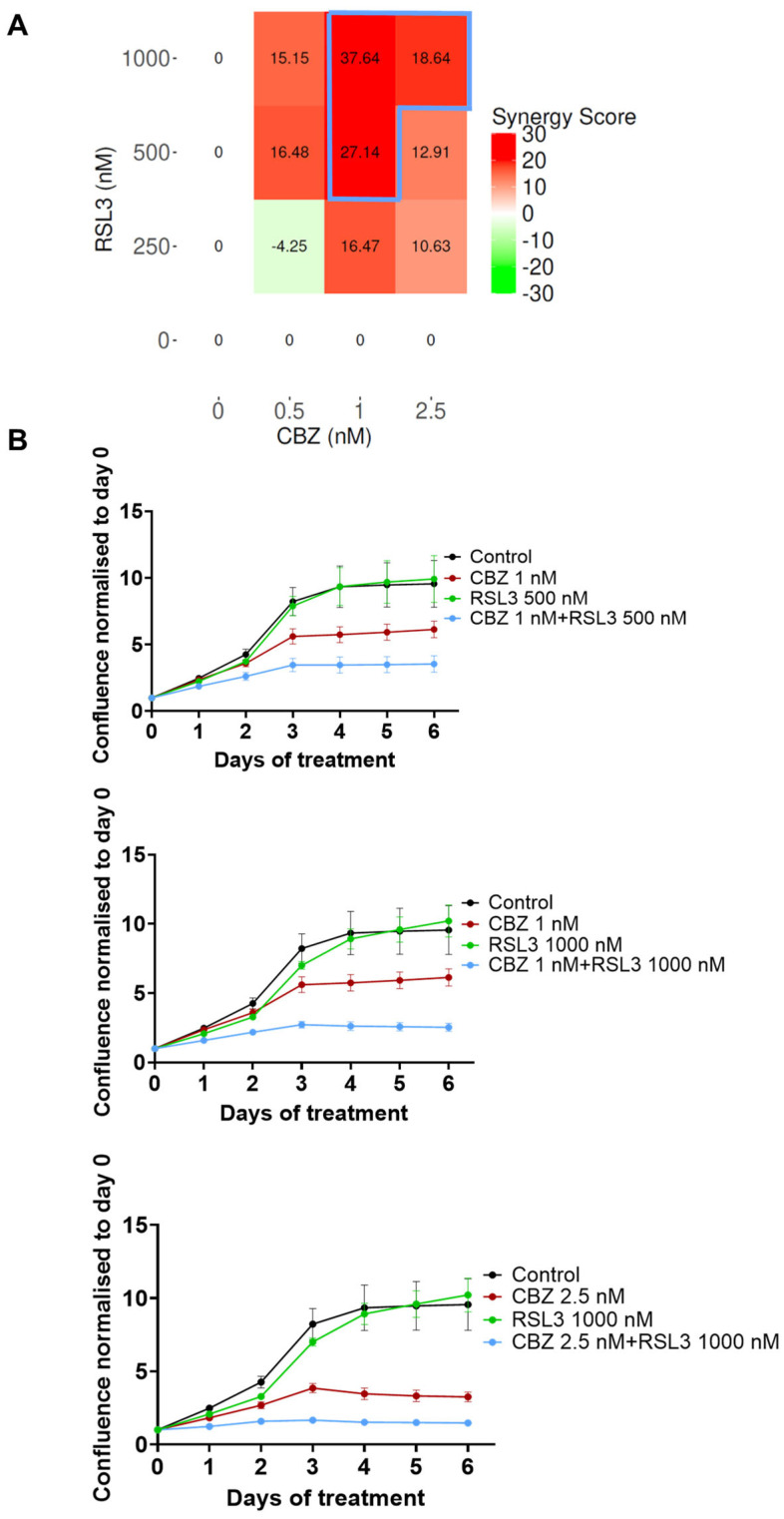
Combinatorial effects of CBZ and RSL3 in MCF-7 cells. MCF-7 cells were treated with different concentrations of CBZ and RSL3 as single drugs or as combinations. Drug responses were evaluated by quantifying cell confluence through real-time Incucyte analysis for up to 6 days. Combinatorial effects were analyzed, and synergy scores were derived using SynergyFinder Plus software. (**A**) Synergy scores of all combinations used, derived by the HSA model, confirm the synergistic effects of certain concentrations. Combinations showing high synergy scores are outlined in blue. (**B**) Plots showing drug responses of combinations that showed synergistic effects and highest scores (outlined in blue in panel (**A**)). Presented here are average values with standard deviations (error bars) of % cell confluence over a period of 6 days from one out of three independent experiments run with triplicates.

The microtubule inhibitor, CBZ, and the ferroptosis inducer, RSL3, are well-known cytotoxic agents and have been utilized in several studies along with other therapeutic agents [15,16,21,22]. Our previous studies have shown that PEBCA NPs are suitable to encapsulate hydrophobic drugs such as CBZ, which also improved the therapeutic effects [27,29]. This prompted us to explore the combinatorial effects of two encapsulated drugs and compare them with the combination of free drugs. Thus, we decided to encapsulate RSL3 into PEBCA NPs and investigate whether a combinatory treatment with RSL3 and CBZ could improve the cytotoxic effects compared to the drugs alone. Studies were performed in four breast cancer cell lines with different origins, molecular characteristics and sensitivity to CBZ or RSL3. The treated cells were monitored for their proliferative capacity, and the combinatorial effects were analyzed using the SynergyFinder Plus application. Figure 2, Figure 3, Figure 4 and Figure 5 show the effect of combinatorial treatment of CBZ and RSL3, either in their free form or encapsulated in PEBCAs in EO771, SKBR3, JIMT-1 and MCF-7 cells, respectively. The results corresponding to each cell line are discussed as below.

**EO771**: Figure 2A represents the synergy scores for combinations of different concentrations of CBZ and RSL3 in EO771 cells. The HSA reference model is found suitable for this cell line because one of the drugs (CBZ) is not inducing any inhibitory effect. The combination of 100 nM RSL3 and increasing concentrations of CBZ shows the highest synergy scores. Figure 2C shows the response curves for the combinations with the most pronounced synergistic effect. It is notable from the dose–response curves of CBZ and RSL3 (Figures S1 and S2) that EO771 cells are insensitive to CBZ at the concentrations studied but are sensitive to RSL3 in a time- and concentration-dependent manner. However, the sensitivity to RSL3 is transient, and the cells regained the capacity to proliferate effectively at a later time point. Hence, the evident combinatorial effects are relevant, which means that CBZ plays a significant role in maintaining the inhibition caused by RSL3, and this inhibition increases with increasing concentration of CBZ. Similar is the case of encapsulated drugs, where PEBCA-CBZ and PEBCA-RSL3 showed similar dose responses (Figures S1 and S2) and combinatorial effects as free drugs (Figure 2C,D).

**SKBR3**: Figure 3A,B show the synergy scores representing the effect of combinatorial treatment by free drugs and encapsulated drugs, respectively, in SKBR3 cells. Differing from EO771 cells, SKBR3 cells are sensitive to both CBZ and RSL3 with increasing concentrations (Figures S1 and S2), and the combination of drugs shows additive effects, where the synergy scores are near to zero. The Bliss reference model was used. The highest scores are shown when 10 nM CBZ was combined with different concentrations of RSL3, and this pattern is similar for both free and encapsulated drugs, as shown in Figure 3A,B. The real-time effect of the combinations showing the highest synergy scores is presented in Figure 3C,D, corresponding to free drugs and encapsulated drugs, respectively.

**JIMT-1**: As shown in Figures S1 and S2, JIMT-1 cells are sensitive to both CBZ and RSL3 in a dose-dependent manner. Hence, the Bliss reference model was chosen. Also, the combined effect of different concentrations of the drugs either in free or encapsulated forms is shown in Figure 4A and 4B, respectively, from which it is obvious that certain combinations seem to induce additive effects (scores 0–10). Plots comparing the effects of these combinations when the drugs are either in their free or encapsulated forms are shown, respectively, in Figure 4C,D. Generally, the trends are similar except for slight differences in the inhibitory effects between free RSL3 and PEBCA-RSL3, which eventually reflect in the synergy scores as well.

**MCF-7**: MCF-7 cells are sensitive to CBZ in a concentration-dependent manner (Figure S1) but resistant to even high concentrations of RSL3 (Figure S2). Hence, assessing combinatorial effects using the HSA model was found appropriate. The resistance to RSL3 in this cell line was correlated to the lack of endogenous acyl-CoA synthetase long-chain family member 4 (ACSL4) [38], which was reported as an essential component to induce ferroptosis [38]. Figure 5A shows the combinatorial effects of different concentrations of CBZ and RSL3, and the synergy scores show that the combination is highly synergistic. It is quite interesting that even if the cells are tolerant to RSL3 alone, this compound/drug/inhibitor sensitizes the cells to chemotherapy. We could not test encapsulated drugs in the case of MCF-7 because we knew from our previous experience [28] that empty PEBCA NPs themselves induce toxic effects in MCF-7 cells at concentrations equivalent to the higher concentrations of encapsulated RSL3. Of note, the concentrations of PEBCA used in the case of all other cell lines were below the toxic range, as is evident from Figure S3.

### 3.3. In Vitro Combinatorial Effects Are Similar for Free Drugs and Encapsulated Drugs

Combining multiple therapeutic drugs simultaneously within a drug delivery system can unify the pharmacokinetic properties of the drugs [39,40]. Moreover, it may offer protection of labile drugs along with controlled release of therapeutics for aiding combinatorial effects [41]. However, there are several challenges that make this strategy less encouraging. Vyxeos, a liposomal formulation of daunorubicin and cytarabine, is the only one that has gained approval so far [42]. Co-encapsulating drugs within proximal 3D space could promote several interactions that may affect the stability and optimal release of the drugs. Moreover, it is difficult to control all the possible interactions and their consequences to achieve optimal co-encapsulation while maintaining batch-to-batch reproducibility [41]. In the present study, the drugs are not co-encapsulated; instead, they are independently loaded within the same type of PEBCA NPs. This is mainly because it is not practical to evaluate the range of combinatorial effects in different cells when the drugs are co-loaded in a single known ratio within the NPs. This approach will also unify the drug delivery properties in the physiological system and would also allow sequential treatment of multiple therapeutics where one drug could sensitize the cells to make them more prone to the effect induced by the other. However, this may result in a higher accumulation of the NPs, and it is therefore important to investigate the safety limit of the NPs. Higher loading efficiency might be one of the criteria that need to be considered. As mentioned before, the present study utilized independently loaded CBZ and RSL3 within PEBCA NPs, and their combinatorial effects were compared with those of free drugs in three of the cell lines studied. The results shown in Figure 2, Figure 3 and Figure 4 demonstrate that the combination of encapsulated drugs is equally effective as the combination of free drugs in all the cell lines in the in vitro conditions, also confirming that encapsulation in PEBCA does not affect the individual drug’s mode of action. This study thus supports investigating the potential for improved efficacy of PEBCA drugs in the in vivo setting and perhaps also including a co-encapsulation strategy for the drug combination.

### 3.4. Combinatorial Effects Are Cell Type Dependent

The four cell lines differ in their sensitivity to single drugs. It is well known that the sensitivity of different cells to ferroptotic agents such as RSL3 depends upon various factors such as the ability of ACSL4 to modulate the composition and distribution of phospholipids [38,43], cell density [44], and the endogenous activity of GPX4 [45]. Similarly, sensitivity to CBZ is also associated with several molecular and cellular factors like activity of the tyrosine kinase receptor protein, Ror2 [46], and expression of the MDR1 gene [47]. From Figure 2, Figure 3, Figure 4 and Figure 5, one could conclude that the combinatorial effects of CBZ and RSL3 are highly cell-dependent. Interestingly, the combination induced highly synergistic effects in EO771 and MCF-7 cells, where the cells have low sensitivity to one of the drugs. This may be caused by mutual sensitization as reported for RSL3, which gives a synergistic effect with many compounds, including taxanes [22,23,24,25,48]. For instance, Ye et al. reported that a low concentration of paclitaxel acts synergistically with RSL3 in hypopharyngeal squamous cell carcinoma by paclitaxel-mediated upregulation of mutant p53 expression, which in turn suppresses the transcription of SLC7A11, rendering the cells more vulnerable to RSL3-induced ferroptosis [49]. Similarly, Yuan et al. reported that RSL3 increases the chemosensitivity of triple-negative breast cancer to paclitaxel by inducing ferroptosis through NF-κB signaling [22]. Hence, single or multiple mechanisms may be involved, which may differ with the molecular characteristics of each cell line.

### 3.5. Cell Viability Assay Correlates with Cell Proliferation Assay

**Figure 6:**
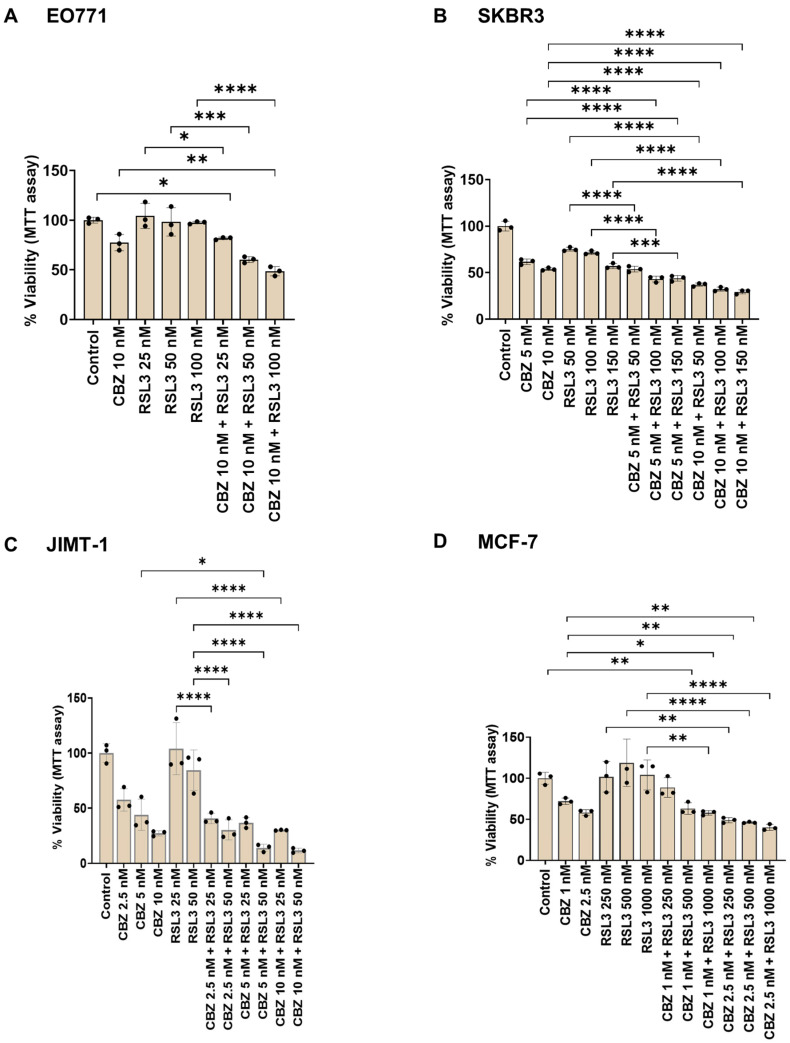
MTT assay showing combinatorial effects of drugs alone in breast cancer cell lines. Cells were treated with drugs alone or their combinations for 96 h, and the cytotoxicity was measured by MTT assay. Data corresponding to the range of drug concentrations that showed combinatorial effects with the highest significance are shown. Data are presented as % cell viability ± standard deviations from one out of three independent experiments, each run in triplicate. * *p* < 0.05, ** *p* < 0.01, *** *p* < 0.001, **** *p* < 0.0001.

**Figure 7:**
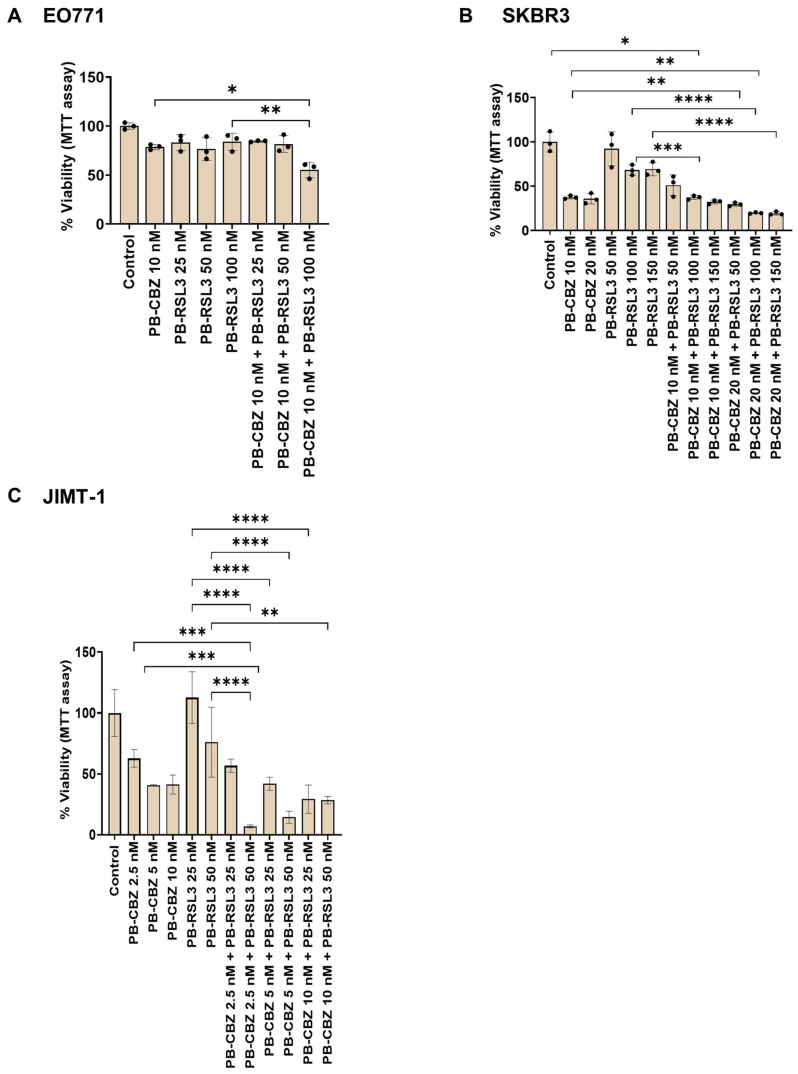
MTT assay showing combinatorial effects of PEBCA drugs in breast cancer cell lines. Cells were treated with PEBCA drugs or their combinations for 96 h, and the cytotoxicity was measured by MTT assay. Data corresponding to the range of PEBCA drug concentrations that showed combinatorial effects with the highest significance are shown. Data are presented as % cell viability ± standard deviations from one out of three independent experiments, each run in triplicate. * *p* < 0.05, ** *p* < 0.01, *** *p* < 0.001, **** *p* < 0.0001. PEBCA is abbreviated to PB in graphs.

Cell proliferation assays evaluate the ability of the cells to divide, whereas cell viability assays assess single or multiple parameters of cellular function in response to treatment [50]. Evaluating drug responses utilizing different methods is valuable and will enhance our understanding of the mechanism of action. Hence, we have also evaluated the combinatorial effects by MTT assay, which assesses the metabolic activity of cells by measuring NAD(P)H-dependent oxidoreduction. Results showing effects corresponding to free drugs and PEBCA drugs are shown in Figure 6 and Figure 7, respectively.

As shown in Figure 6, certain combinations induced improved cytotoxic effects compared to the single drugs in all cell lines studied. It is important to note that the concentrations that showed evident combinatorial effects differ between the two assays, especially in EO771 cells. This might be because the MTT assay was assessed after 96 h and could not be continued because the cells became confluent. In contrast, the proliferation assay was continued up to 6 days, which was essential to distinguish the combinatorial effects from the transient inhibitory effect of RSL3 alone. In addition to this, the comparison between single drugs and combinations is also different in the two assays, which might be due to differences in the end points and assessed parameters. Similar patterns were observed in the case of PEBCA drugs or their combinations (Figure 7). Altogether, it can be concluded that the combinatorial effects evaluated by the two methods correlate, which confirms that the drug combinations are effective.

### 3.6. Combinatorial Treatment Did Not Cause Immediate Effects on ROS Levels

ROS molecules exhibit pleiotropic functions within the cell. Besides modulating cellular signaling, their excess also induces DNA damage, protein oxidation or lipid peroxidation, being responsible for different cell death mechanisms [51]. Increased ROS levels upon CBZ and RSL3 treatment have been reported in several studies. CBZ was described to have pro-oxidant effects and mediate apoptosis in cancer cells by increasing ROS and mitochondrial damage [52]. Kosaka et al. reported decreased expression of the Sestrin 3 gene leading to elevated ROS levels upon CBZ treatment in cancer cells [53]. RSL3 covalently binds to the antioxidant protein GPX4, and there are several papers reporting increased levels of ROS after RSL3 treatment [21]. GPX4 is considered a central player in regulating ferroptosis, as this protein removes lipid peroxides that are produced from polyunsaturated fatty acids by elevated ROS levels.

To investigate whether the combinatorial effects are mediated through ROS production, the ROS levels were assessed using the fluorescence probe CM-H_2_DCFDA after treating the cells for up to 5 h. As shown in Figure S4, no effects on ROS production were induced by free or encapsulated CBZ and RSL3, nor their combinations, in all the cell lines studied, irrespective of their differences in H_2_O_2_-induced ROS production (Figure S5). However, we also observed that empty PEBCA NPs caused a slight increase in ROS levels in SKBR3 cells at the equivalent concentrations of PEBCA drugs used for the studies (Figure S6). We have earlier published that treatment with higher concentrations of empty PEBCA NPs (25 µg/mL) increased ROS production in MDA-MB-231 cells [54], whereas the concentrations used in the present studies are below 2 µg/mL. Hence, we speculate that this effect of empty PEBCA is cell dependent. To conclude, the combinatorial treatment did not cause an increase in ROS production at an early time point in any of the cell lines.

### 3.7. Combinatorial Effects Are Not Accompanied by Immediate Effects on Cell Metabolism

**Figure 8:**
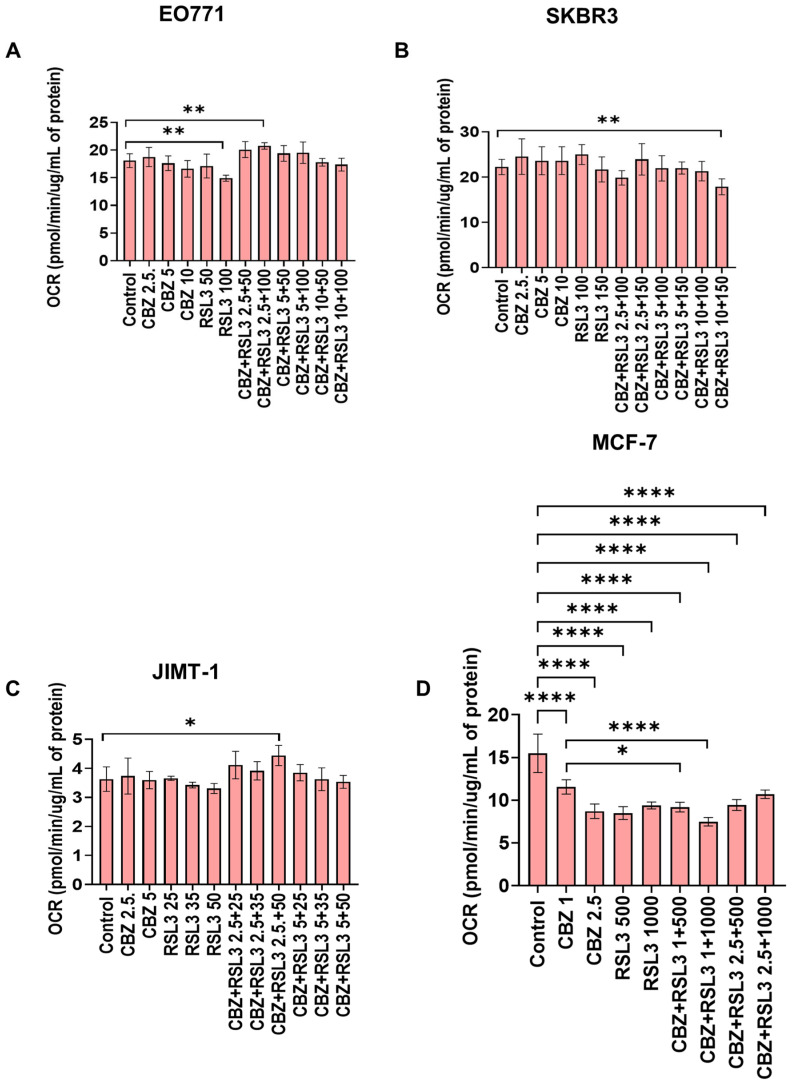
Effect of free drugs or combinations on mitochondrial respiration. Cells were treated for 5 h at 37 °C and were analyzed by Mito Stress Assay using a Seahorse analyzer. Shown here are data from (**A**) EO771, (**B**) SKBR3, (**C**) JIMT-1 and (**D**) MCF7 cells. The graphs show variation of oxygen consumption rate (OCR) after addition of different modulators of mitochondrial respiration in terms of maximal respiration. Values represent the mean ± SD of three technical replicates. One representative experiment is shown. * *p* < 0.05, ** *p* < 0.01, **** *p* < 0.0001.

**Figure 9:**
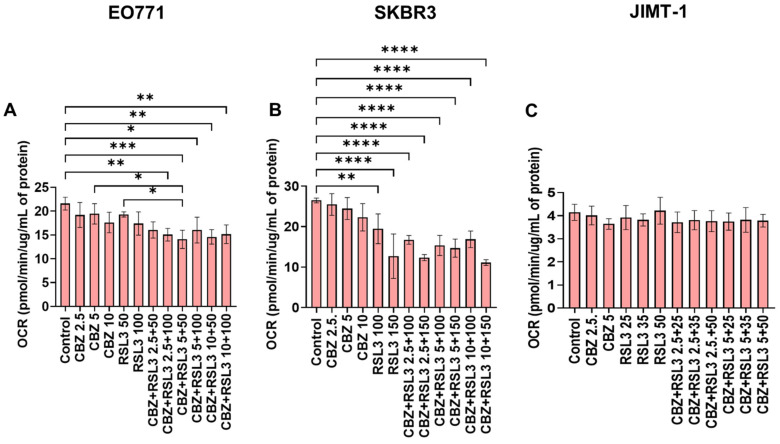
Effect of PEBCA drugs or their combinations on mitochondrial respiration. Cells were treated for 5 h at 37 °C and were analyzed by Mito Stress Assay using a Seahorse analyzer. Shown here are data from (**A**) EO771, (**B**) SKBR3 and (**C**) JIMT-1 cells. The graphs show variation of oxygen consumption rate (OCR) after addition of different modulators of mitochondrial respiration in terms of maximal respiration. Values represent the mean ± SD of three technical replicates. One representative experiment is shown. * *p* < 0.05, ** *p* < 0.01, *** *p* < 0.001, **** *p* < 0.0001.

We investigated whether the combinatorial effects can be correlated with any changes in cell metabolism. Hence, we studied the effect of free drugs, PEBCA drugs and combinations on mitochondrial metabolism using a Seahorse Analyzer employing a MitoStress assay. Data are presented as oxygen consumption rate (OCR) in terms of basal respiration (Figures S7 and S8) and maximal respiration (Figure 8 and Figure 9). As shown in Figure 8A and Figure S7A, treatment with a higher concentration of RSL3 caused a significant decrease in OCR in EO771 cells. Otherwise, the free drugs or combinations did not induce any large effects. Nevertheless, in the case of PEBCA drugs, a gradual decrease in OCR was observed towards higher concentrations of the combinations (Figure 9A and Figure S8A). The decrease is caused mainly by the effect of the encapsulated drugs, as the empty PEBCA NPs had no effect (Figure S9A) on the cell line. The effect of RSL3 fits with our recently published data for MDA-MB-231 cells [55]. Others have also shown that GPX4 inhibition with RSL3 increased ROS levels in mitochondria, leading to their impaired function and decrease in maximal respiration [56]. Moreover, decreased OCR for both basal metabolism and maximal respiration shows that cellular metabolism is affected by the treatment with CBZ and RSL3. This might be due to a decreased mitochondrial potential (ΔΨ_Μ_) caused by RSL3 [55,57]. Moreover, it has been reported that CBZ also decreases ΔΨ_Μ_, i.e., giving mitochondrial depolarization in prostate cancer cells due to increased ROS levels [52]. Hence, we speculate that drugs and NPs together contribute to the effect caused by PEBCA drugs.

JIMT-1 and SKBR3 cell lines did not show significant responses to free drugs or combinations, except that the higher concentrations of combinations significantly decreased the OCR in SKBR3 cells (Figure 9B and Figure S7B for SKBR3 cells and Figure 9C and Figure S7C for JIMT-1 cells, respectively). At the same time, these cell lines responded differently to the treatment with PEBCA drugs and combinations. The OCR in SKBR3 cells was significantly affected by PEBCA-RSL3 and all of the combinations (Figure 9B and Figure S8B), while JIMT-1 cells seemed unaffected (Figure 9C and Figure S8C). It is interesting to note that empty PEBCA also induced a decrease in OCR in SKBR3 cells and JIMT-1 cells at the highest concentrations equivalent to the combinations (Figure S9B and S9C , respectively). This did, however, not seem to have a profound impact on the effect of PEBCA drugs in JIMT-1 cells, as shown in Figure 9C and Figure S8C. Whereas in SKBR3 cells, when comparing Figure 9B, Figures S8B and Figure S9B, we could conclude that both RSL3 and PEBCA NPs are responsible for the action. It should be studied whether CBZ also contributes to this effect when it is combined with RSL3 and PEBCA. Concurrently, the effects of empty PEBCA NPs on SKBR3 cells are so evident, as the results from the ROS assay (Figure S6B) correlate well with those of the Seahorse assay (Figure S9B). We already showed that empty PEBCA NPs increase ROS accumulation in MDA-MB-231 cells at a concentration of 25 µg/mL [54]. In the present study, even though we used several-fold lower concentrations of NPs, SKBR3 cells might be more susceptible to NP-induced ROS production than MDA-MB-231 cells. However, none of these effects were reflected in the proliferation and cell viability assays, where free and PEBCA drugs showed similar patterns (Figure 3, Figure 6C and Figure 7C). This is supported by our previous report, where MS-based proteomics studies revealed that different breast, colon and prostate cancer cell lines have altered proteomes upon NP treatment [28]. Here, treatment with PEBCA-CBZ led to a decrease in the total amount of proteins, even though it was not reflected in the cytotoxicity pattern of free and encapsulated CBZ in all of the three cell lines studied [28]. MCF-7 cells, on the other hand, are resistant to RSL3. Still, all concentrations of RSL3, CBZ and the combinations caused a significant decrease in OCR in these cells, as shown in Figure 8D and Figure S7D. However, there were no differences between drugs alone and combinations.

Altogether, the results from the four cell lines emphasize that the combinatorial effects are not due to immediate changes in cell metabolism as measured with the Seahorse Analyzer. However, it is possible that the treatment may induce metabolic changes after long-time exposure, which has to be explored further. Also, it is evident that PEBCA NPs alone or in combination with the drugs could influence the ROS production and cell metabolism in a cell-dependent manner, even though these changes may not be reflected in their cytotoxic effects when compared to the free drugs in the in vitro setting.

## 4. Conclusions

We here demonstrate that CBZ and RSL3 can give synergistic effects in vitro, indicating the possibility of developing combinatorial nanomedicine using these drugs. The combinatorial effects were similar for the free and encapsulated drugs. Remarkably, synergy was obtained even in cell lines that are non-responsive to monotreatment with one of the compounds. The drug combination did not cause immediate effects on ROS production and cell metabolism. However, we cannot exclude the possibility of an impact after a prolonged period. The fact that also encapsulated drugs give synergy could be important for the possible in vivo use, as this may provide the drug with a better pharmacokinetic profile, as documented in our previous in vivo studies with PEBCA-CBZ [27,29]. Based on the promising results shown here, future studies should address the mechanisms behind the combinatorial effects in different cell types. Importantly, drug combinations should be tested in preclinical tumor models.

## Data Availability

All data supporting the findings are shown in the paper, and further queries can be directed to the corresponding author upon reasonable request.

## Supplementary Materials

The following supporting information can be downloaded at https://www.mdpi.com/article/10.3390/pharmaceutics17050657/s1.
