# Supplementary material for: Combinatorial Effects of Free and Nanoencapsulated Forms of Cabazitaxel and RAS-Selective Lethal 3 in Breast Cancer Cells
Source: Pharmaceutics. 2025 May 17;17(5):657. doi: 10.3390/pharmaceutics17050657 (PMC12115222; doi:10.3390/pharmaceutics17050657)
Supplement: Supplementary file 1 [file pharmaceutics-17-00657-s001.zip › pharmaceutics-3608454-supplementary.pdf]

Combinatorial effects of free or encapsulated forms of Cabazitaxel with RSL3- loaded nanoparticles in breast cancer cells

Remya Valsalakumari[1,2#], Marek Feith[1,3#], Solveig Pettersen[1], Andreas Åslund[6], Yrr Mørch[6], Tore Skotland[2,4], Kirsten Sandvig[2,4,5], Gunhild Mari Mælandsmo[1,6], Tore-Geir Iversen[2,4]

[1]Department of Tumor Biology, Institute for Cancer Research, Oslo University Hospital, 0379 Oslo, Norway

[2]Department of Molecular Cell Biology, Institute for Cancer Research, Oslo University Hospital, 0379 Oslo, Norway

[3]Institute of Clinical Medicine, Faculty of Medicine, University of Oslo, 0379, Oslo, Norway

[4]Centre for Cancer Cell Reprogramming, Faculty of Medicine, University of Oslo, 0379 Oslo, Norway

[5]Department of Biosciences, University of Oslo, 0316 Oslo, Norway

[6]Department of Biotechnology and Nanomedicine, SINTEF AS, Trondheim, 7034, Norway

[7]Department of Medical Biology, University of Tromsø, 9019 Tromsø, Norway

#Contributed equally

Correspondence: Tore-Geir Iversen, Department of Molecular Cell Biology, Institute for Cancer Research, Oslo University Hospital, 0379 Oslo, Norway.

Email: t.g.iversen@ous-research.no

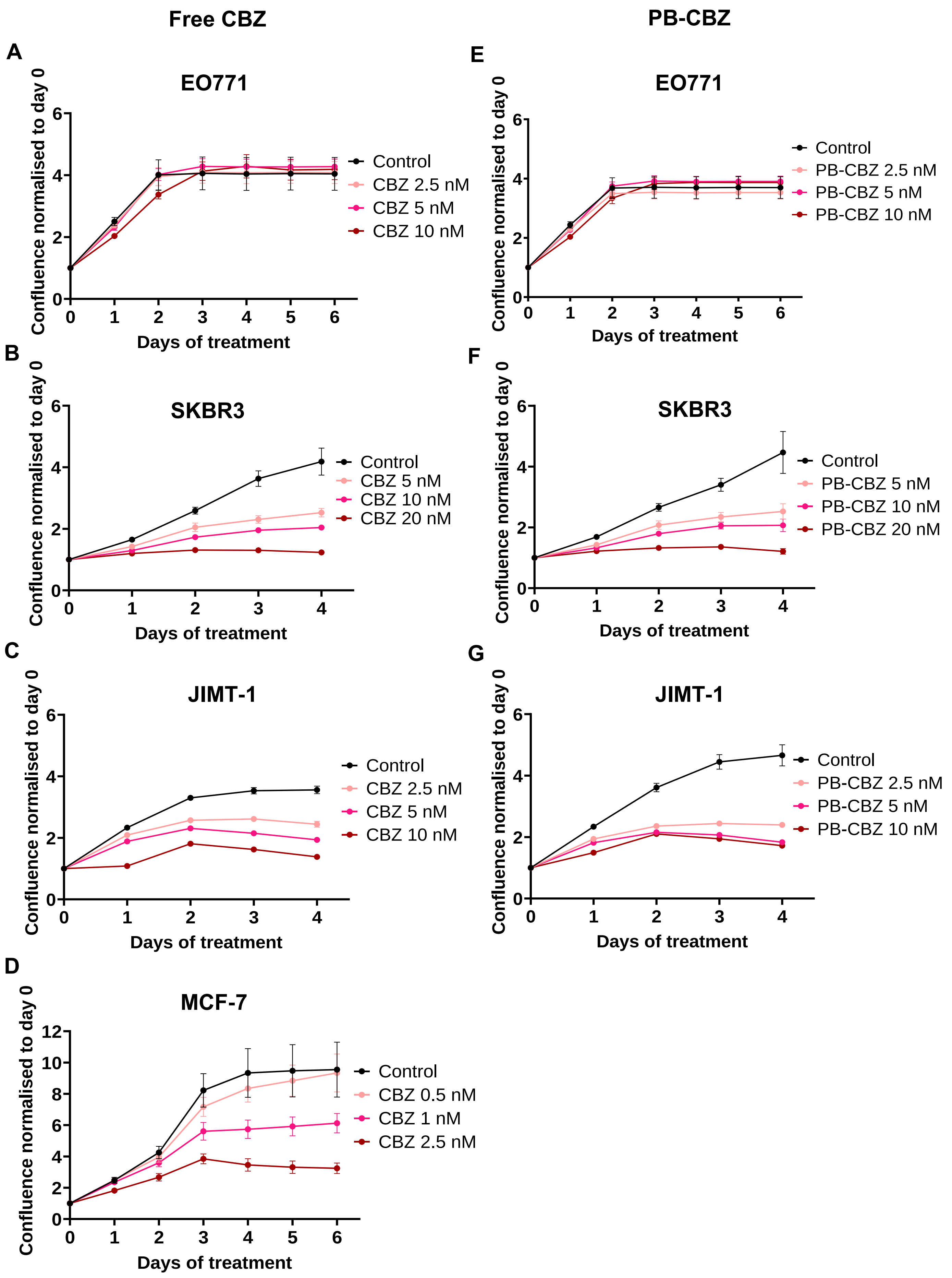

Figure S1. Dose response curves for CBZ and PB-CBZ in breast cancer cell lines. Cells were treated and drug responses were evaluated by quantifying cell confluence through real time Incucyte analysis for up to 4-6 days.

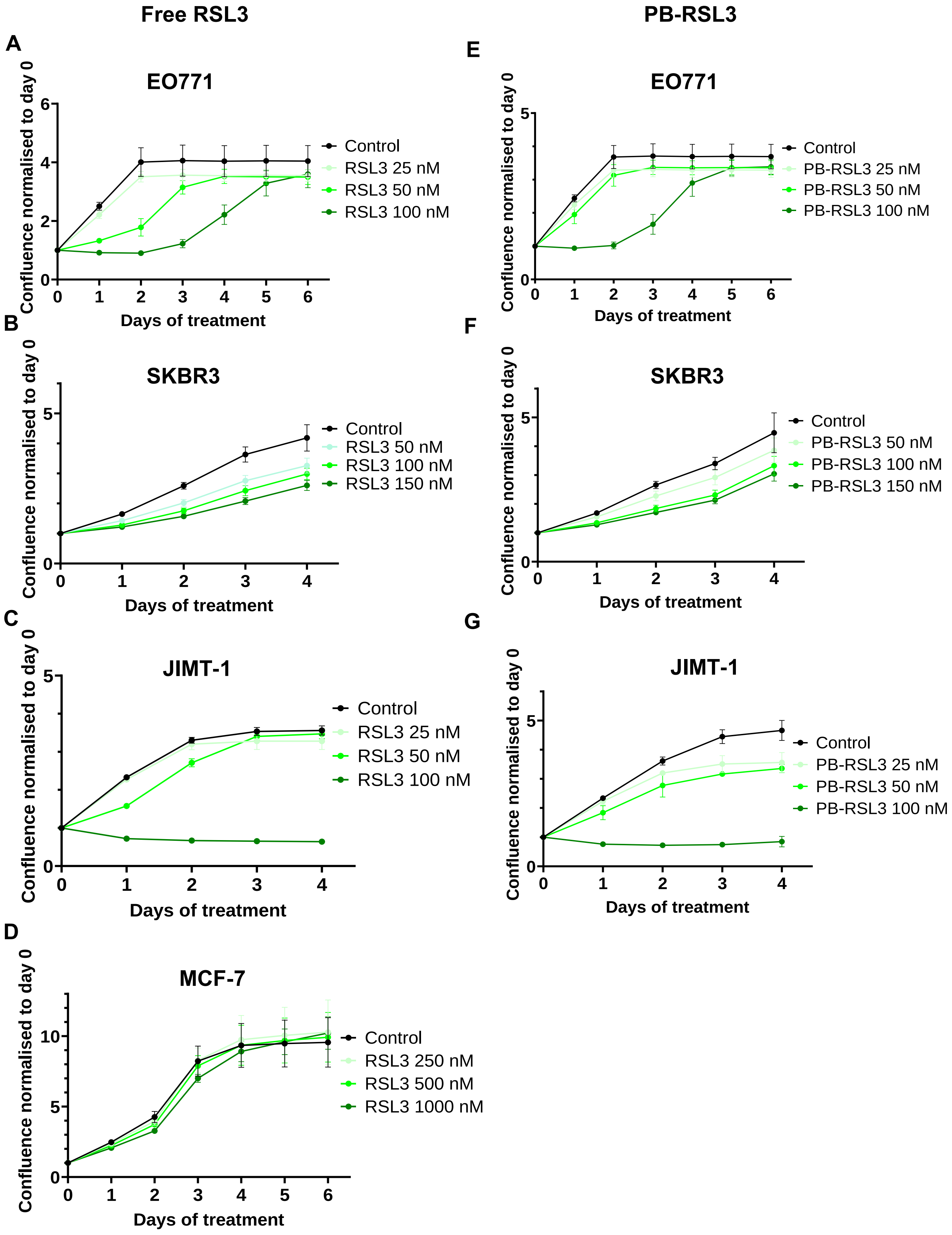

Figure S2. Dose response curves for RSL3 and PB-RSL3 in breast cancer cell lines. Cells were treated and drug responses were evaluated by quantifying cell confluence through real time Incucyte analysis for up to 4-6 days.

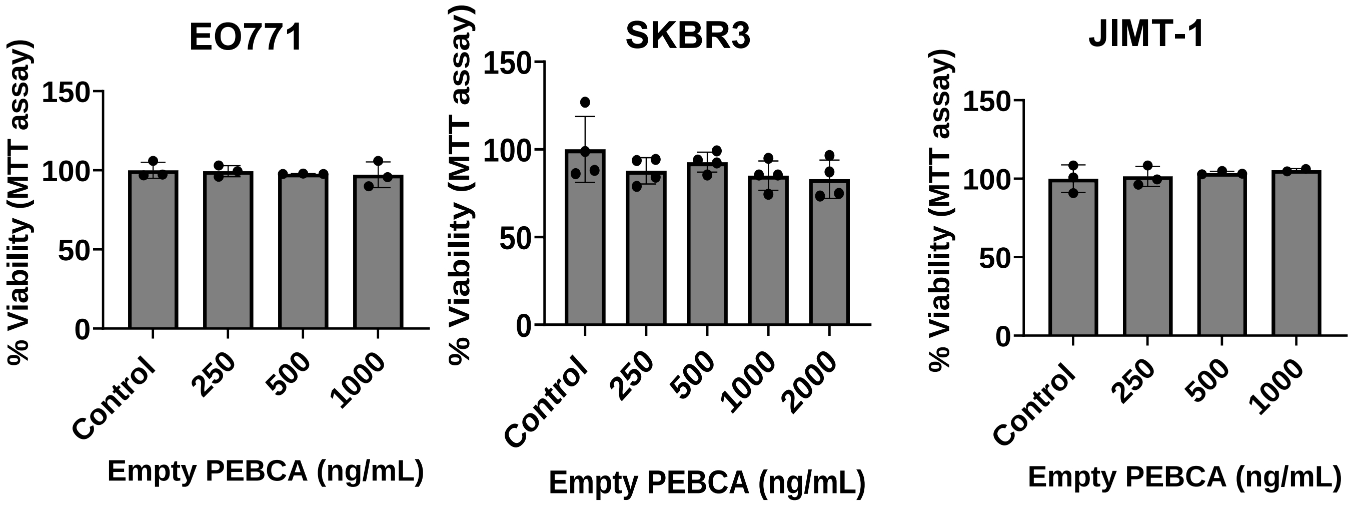

Figure S3. Effect of empty PEBCA on cell viability. Cells were treated with different concentrations of empty PEBCA NPs for 96 hr and the cell viability was assessed by MTT assay. Bars represent mean ± SD from one experiment, run with triplicates.

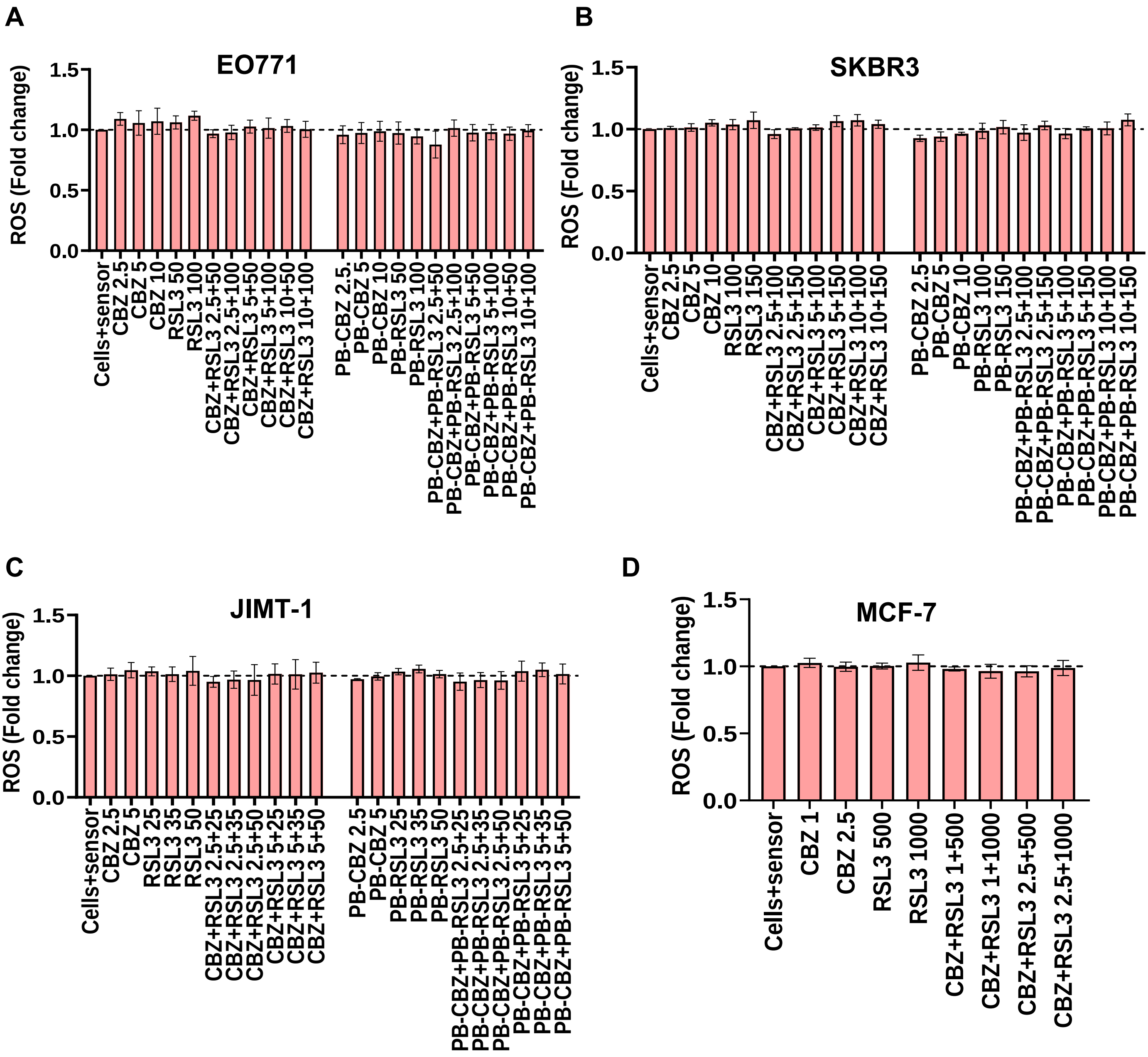

Figure S4. Effect of treatments on ROS levels in breast cancer cell lines. Cells were treated with either drugs alone or PEBCA drugs or combinations for 5 hr at 37 °C and intracellular ROS levels were estimated by using fluorescence based CM-H_2DCFDA probe. Shown here are data from A) EO771, B) SKBR3, C) JIMT-1 and D) MCF7 cells. Bars represent mean ± SEM from three independent experiments.

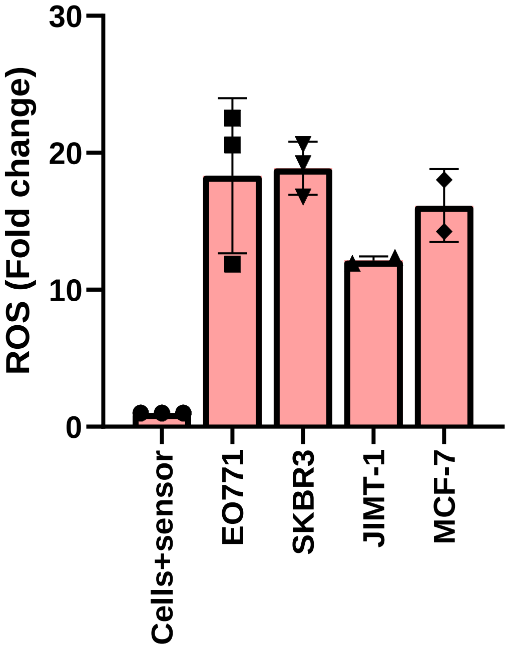

Figure S5. ROS levels in cell lines when treated with H_2O_2. Cells were treated with 500 μM of H_2O_2 for 1 hr at 37 °C and intracellular ROS levels were estimated by using fluorescence based CM-H_2DCFDA probe.

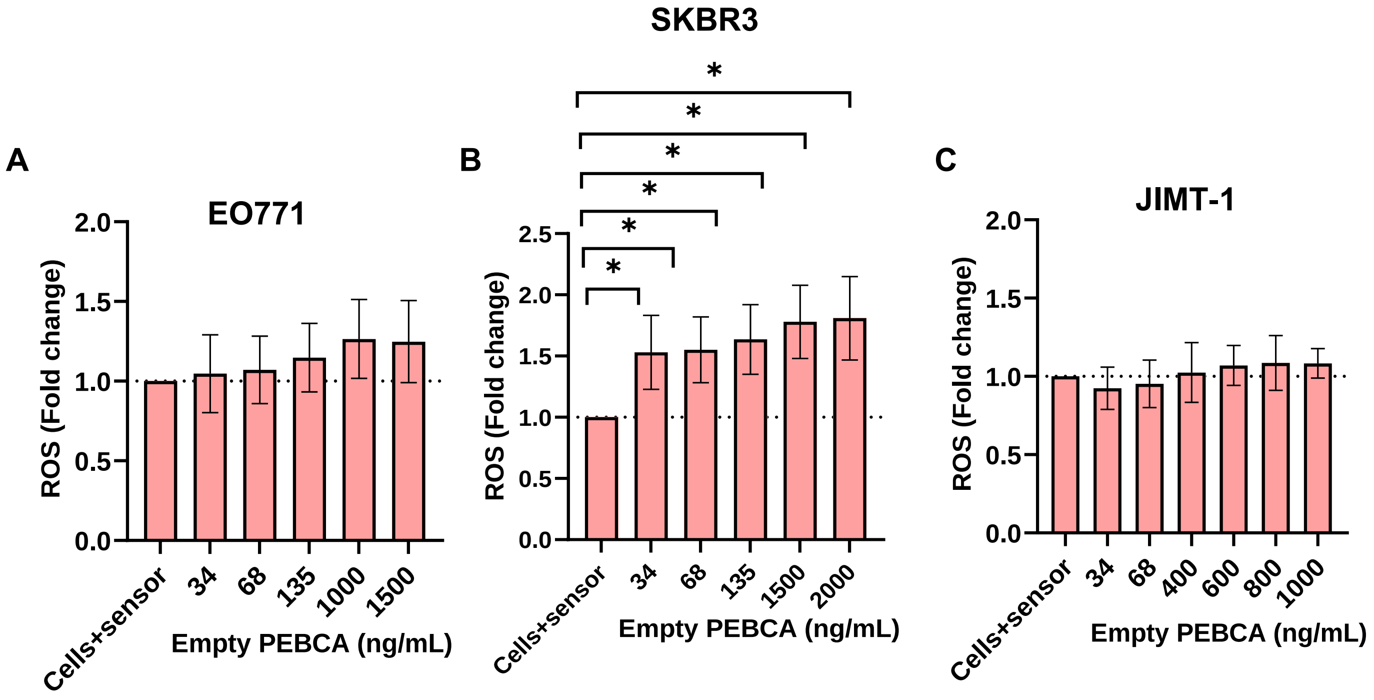

Figure S6. Effect of empty PEBCA on ROS production after 5 hr of treatment. Cells were treated with different concentrations of empty PEBCA NPs for 5 hr at 37 °C and intracellular ROS levels were estimated by using fluorescence based CM-H_2DCFDA probe. Bars represent mean ± SD from two independent experiments, each run in triplicates.

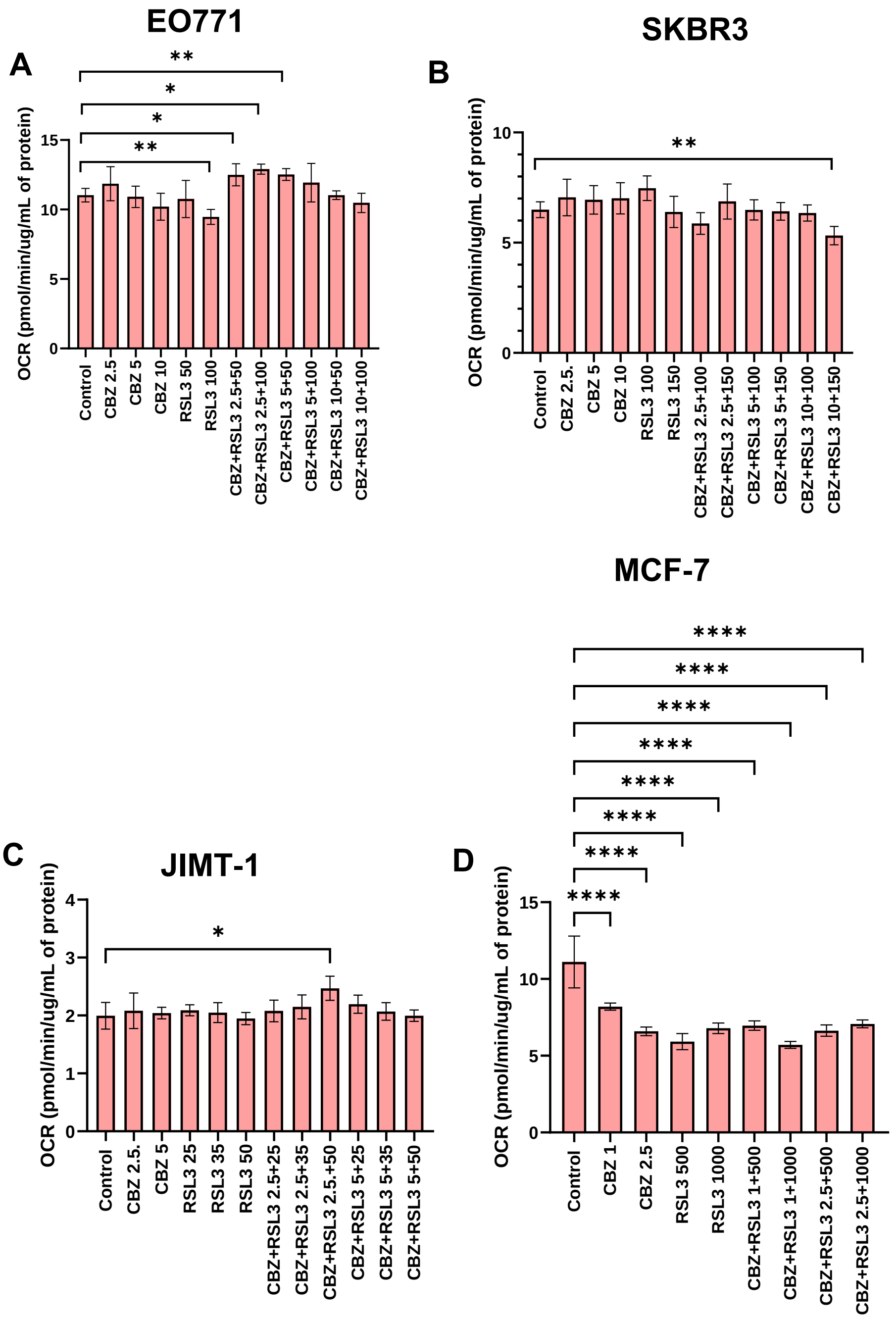

Figure S7. Effect of free drugs or combinations on mitochondrial respiration. Cells were treated for 5 hr at 37 °C and were analyzed by Mito Stress Assay using Seahorse analyzer. Shown here are data from A) EO771, B) SKBR3, C) JIMT-1 and D) MCF7 cells. The graphs show variation of oxygen consumption rate (OCR) after addition of different modulators of mitochondrial respiration in terms of basal respiration. Bars represent mean ± SD of three technical replicates. One representative experiment is shown.

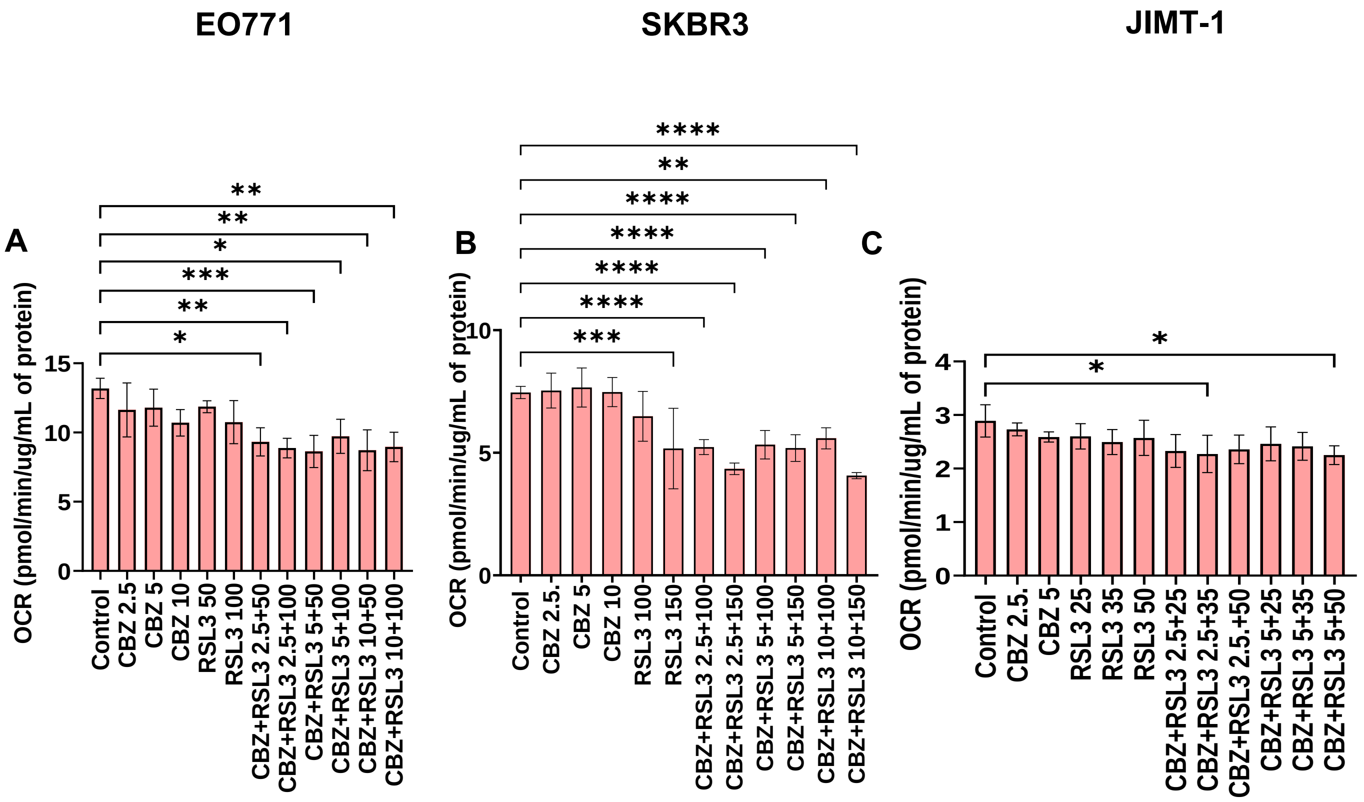

Figure S8. Effect of PB-drugs or combinations on mitochondrial respiration. Cells were treated for 5 hr at 37 °C and were analyzed by Mito Stress Assay using Seahorse analyzer. Shown here are data from A) EO771, B) SKBR3 and C) JIMT-1 cells. The graphs show variation of oxygen consumption rate (OCR) after addition of different modulators of mitochondrial respiration in terms of basal respiration. Bars represent the mean ± SD of three technical replicates. One representative experiment is shown.

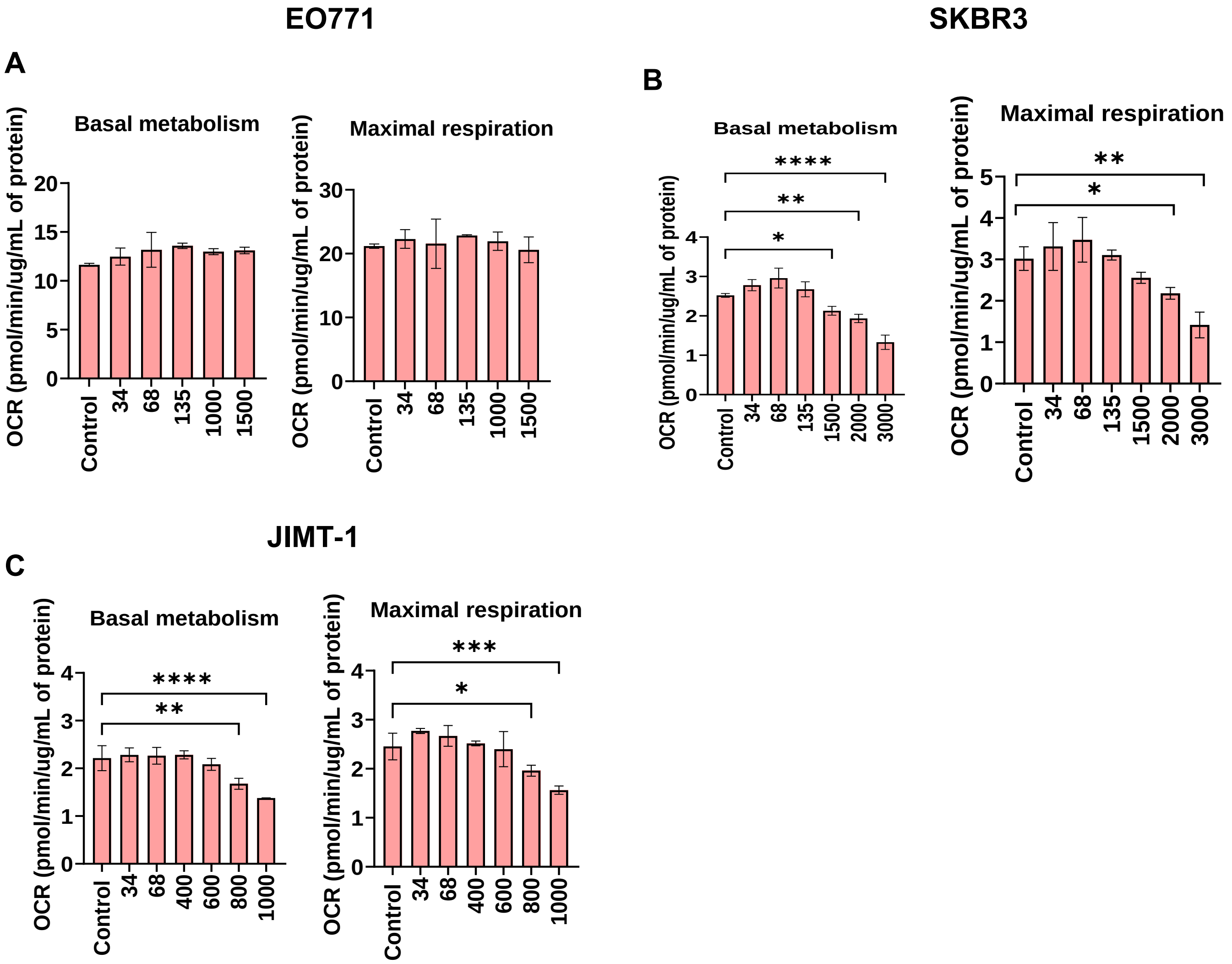

Figure S9. Effect of empty PEBCA on mitochondrial respiration. Cells were treated fwith empty PEBCA NPs at an equivalent concentration of encapsulated NPs 5 hr at 37 °C and were analyzed by Mito Stress Assay using Seahorse analyzer. Shown here are data from A) EO771, B) SKBR3 and C) JIMT-1 cells. The graphs show variation of oxygen consumption rate (OCR) after addition of different modulators of mitochondrial respiration in terms of maximal respiration. Points represent the mean ± SD of three technical replicates. One representative experiment is shown.
